# Clinical Outcomes and Prognostic Factors in Metastatic Triple-Negative Breast Cancer: A Real-World Data Analysis

**DOI:** 10.14740/wjon2713

**Published:** 2026-03-05

**Authors:** Moe Itakura, Yoshiya Horimoto, Yumiko Ushiyama, Yuko Ueki, Yumiko Ishizuka, Yoichi Koyama, Kyoko Orimoto, Hiroki Kusama, Takahiko Kawate, Takashi Ishikawa, Junichiro Watanabe, Goro Kutomi

**Affiliations:** a Department of Breast Oncology, Juntendo University Faculty of Medicine, Tokyo, Japan; b Department of Human Pathology, Juntendo University Faculty of Medicine, Tokyo, Japan; c Department of Breast Surgery and Oncology, Tokyo Medical University, Tokyo, Japan

**Keywords:** Triple-negative breast cancer, Metastasis, Overall survival, Chemotherapy, Postoperative surveillance

## Abstract

**Background:**

Metastatic triple-negative breast cancer (mTNBC) is associated with poor outcomes, and therapeutic strategies remain challenging. This study analyzed real-world data to clarify clinical characteristics, treatment patterns, and survival outcomes, focusing on treatment feasibility at metastatic diagnosis.

**Methods:**

A retrospective analysis was conducted on 96 Japanese women who developed distant metastasis after curative surgery and met the inclusion criteria for the final analysis. Patients who were unable to receive drug treatment due to poor condition were defined as the non-treated group and compared with the treated group, who received at least 4 weeks of systemic therapy. Overall survival was assessed using the Cox proportional hazard model.

**Results:**

Overall, 31% of patients could not receive systemic treatment due to poor condition. The non-treated group was more frequently diagnosed after presenting with symptoms and had a higher prevalence of poor performance status and brain metastasis (P < 0.001). Among the 66 treated patients, the median overall survival was 14 months with an average of 2.2 treatment lines. In exploratory Cox analyses, the number of metastatic organs and treatment with paclitaxel plus bevacizumab were associated with overall survival. Several patients experienced prolonged treatment with oral 5-fluorouracil.

**Conclusions:**

A significant proportion of patients were diagnosed with mTNBC after symptom onset, limiting therapeutic intervention. Diagnosing mTNBC before symptomatic deterioration may expand treatment opportunities. Prospective evaluation of follow-up strategies and biomarkers is required, and further research should clarify treatment positioning, including paclitaxel plus bevacizumab and maintenance therapy with oral agents for biologically low-grade cases.

## Introduction

Triple-negative breast cancer (TNBC) is the most aggressive subtype with the poorest prognosis among breast cancers. To improve patient outcomes, extensive research and drug development have been conducted and as a result, several novel drugs, such as immune-checkpoint inhibitors (ICIs), have recently been introduced. However, there are still many challenges that need to be addressed [1, 2]. Once breast cancer metastasizes, regardless of its subtype, obtaining a cure becomes extremely difficult. Furthermore, patients with metastatic TNBC (mTNBC) have extremely limited overall survival (OS), reportedly ranging from 8 to 13 months [3], and the 5-year relative survival rate is approximately 4–20% [4].

Historically, anthracyclines and taxanes have long been regarded as the standard first-line treatment options for mTNBC [3]. However, in many cases, these drugs have already been used during the perioperative period. In such cases, resistance to these drugs is anticipated, and the effective options are limited. More recently, several novel systemic therapies have been introduced, including ICIs in combination with chemotherapy for programmed death-ligand 1 (PD-L1)-positive mTNBC [5, 6], olaparib for patients with germline *BRCA* mutations [7], and trastuzumab deruxtecan (T-DXd) for those with human epidermal growth factor receptor 2 (HER2)-low tumors [8]. These advances have expanded therapeutic options for selected patient subgroups. However, in the absence of these biomarkers, it remains unclear which chemotherapy agents should be chosen from the available options, such as capecitabine, eribulin, or vinorelbine [9–11]. As mTNBC often progresses rapidly, selecting optimal therapy is challenging.

As such, there is an unmet need for new therapeutic options for mTNBC resistant to anthracyclines and taxanes. While second-generation treatments, such as sacituzumab govitecan (SG), have recently emerged [12, 13], their real-world data (RWD) remain limited. This underscores the importance of analyzing the pre-second-generation treatment era, as done in this study. Naturally, future comparisons with second-generation therapies will also be necessary. Therefore, the aim of this study was to clarify real-world patterns of diagnosis, treatment initiation, and clinical outcomes in patients with mTNBC in the pre-SG, ICI, and T-DXd era. In particular, we evaluated the presence and frequency of patients for whom systemic therapy was not feasible at the time of metastatic diagnosis, based on real-world clinical data. We further compared the clinical circumstances of symptomatic versus asymptomatic detection of metastasis and explored factors associated with treatment feasibility and OS in an exploratory manner.

## Materials and Methods

### Institutional Review Board approval

All procedures performed in studies involving human participants were in accordance with the ethical standards of the Ethics Committee of Juntendo University Hospital and Tokyo Medical University Hospital (approval number: E23-0184).

### Ethical compliance with human study

This study was conducted in compliance with the ethical standards of the responsible institution on human subjects as well as with the Helsinki Declaration.

### Patients

**Figure 1:**
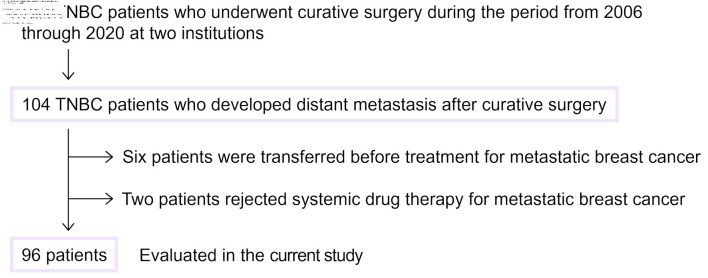
Flowchart of eligible patients. Patient selection criteria for the current study are shown. TNBC: triple-negative breast cancer.

In total, there were 673 patients with TNBC who underwent curative surgery during the period from 2006 through 2023 at Juntendo University Hospital (Tokyo, Japan) and Tokyo Medical University Hospital (Tokyo, Japan). Patients with *de novo* stage IV disease were not included, as this study focused on patients who developed distant metastasis after curative surgery. Among these, 104 patients developed distant metastasis during the follow-up period. Six patients who were transferred to another hospital for treatment at the time of mTNBC diagnosis were excluded from the analysis. Another two patients who rejected systemic drug therapy were also excluded. Hence, data from 96 patients, all Japanese women, were investigated in the current study. Patient selection criteria for the current study are shown in Figure 1.

### Pathological assessment

Biopsy and surgical specimens of primary tumors were pathologically examined and classified based on the World Health Organization classification of tumors for breast cancer, fifth edition. Tumor grade was judged based on nuclear grading from the modified Nottingham histologic score system [14]. TNBC was defined as tumors that tested negative for estrogen and progesterone receptors (less than 1% of tumor cell nuclei staining) and negative for HER2 based on the American Society of Clinical Oncology/College of American Pathologists guidelines [15].

### Systemic drug treatment

First, the non-treated group was defined as patients whose general condition was so poor that they could not receive any systemic drug treatment for mTNBC or could only be treated for less than 4 weeks. Their clinicopathological factors were then compared with the treated group, which received at least 4 weeks of drug treatment. A line of therapy was defined on a regimen basis. Advancement to a new line of therapy was defined as a change in the anticancer agent(s). When analyzing the details of each treatment, the duration of treatment was defined using time to treatment discontinuation (TTD). Patients were followed from the diagnosis of mTNBC until death or the last clinical contact. For time-to-event analyses, the index date was defined individually for each patient as the date of diagnosis of mTNBC. All clinical data were collected up to June 30, 2023, which served as the administrative cut-off date for this study.

### Statistical analysis

JMP 14.2 statistical software (SAS Institute, Inc., Cary, NC) was used for statistical analyses. For comparisons of mean values, unpaired *t*-tests were employed. Comparison between the two groups in contingency tables was conducted employing Fisher’s test. TTD was analyzed as a time-to-event outcome, defined as the interval from treatment initiation to treatment discontinuation for any reason, with ongoing treatments censored at the last follow-up. For comparisons of continuous variables presented as medians, the Mann–Whitney U test was used. For analysis of OS, a Cox proportional hazard model was applied. For the full-model analysis, we selected variables based on their known clinical significance. Given the limited sample size, this multivariable Cox analysis was performed in an exploratory manner. We chose age, tumor grade, disease-free interval, number of metastatic sites, the presence of brain metastasis, baseline neutrophil-to-lymphocyte ratio (NLR), and administration of a combination of paclitaxel (PTX) and bevacizumab (BVZ). Kaplan–Meier curves were calculated for OS and TTD, and the log-rank test was applied for comparisons of survival distributions between patient groups. A P value < 0.05 was considered to indicate a significant difference.

## Results

### Differences between the treated and non-treated groups

Clinicopathological features were compared between the treated group and the non-treated group (Table 1). A significantly higher proportion of patients in the non-treated group had some symptoms, such as dizziness, which triggered detection of mTNBC, compared to the treated group (P = 0.005). The majority of the treated group were diagnosed through routine examinations, whereas significantly more patients in the non-treated group were diagnosed due to the presence of some symptoms. In other words, the majority of patients with some symptoms belonged to the non-treated group (18 out of 35 patients). Thirteen of the 96 patients (14%), all in the non-treated group, had a performance status ≥ 2 at diagnosis of mTNBC, so poor performance status was significantly more common in the non-treated group (P < 0.001). Moreover, there were significantly more brain metastases in the non-treated group, compared to the treated group (P < 0.001). Meanwhile, there were no differences in other factors, such as age, tumor grade, or disease-free interval. Furthermore, among the 86 patients who had received adjuvant chemotherapy, 68 patients received both anthracycline- and taxane-based regimens, nine patients received anthracycline alone, and five patients received taxane alone. The remaining four patients were treated with oral fluoropyrimidine (5-fluorouracil (5-FU)). Among the 68 patients who had received perioperative chemotherapy, a comparison between the treated and non-treated groups revealed that patients in the non-treated group were more likely to have received less intensive chemotherapy (P = 0.003) (Supplementary Material 1, wjon.elmerpub.com).

**Table 1:** Comparisons in Clinicopathological Features Between the Treated and the Non-Treated Groups

Variables	Total (n = 96)	Treated group (n = 66)	Non-treated group (n = 30)	P value
Age^a^ (mean, year) (range)	57 (26–84)	54 (26–84)	59 (29–84)	0.068
Histology				
IDC (NST)	86 (90%)	60 (90%)	26 (87%)	0.528
Others	10 (10%)	6 (10%)	4 (13%)	
pT (mean, mm) (range)	36 (0–125)	37 (0–125)	34 (0–115)	0.721
pN				
Positive	53 (55%)	38 (58%)	15 (50%)	0.489
Negative	43 (45%)	28 (42%)	15 (50%)	
Tumor grade				
High	47 (49%)	31 (47%)	16 (53%)	0.579
Low/intermediate	42 (44%)	30 (45%)	12 (40%)	
Not documented	7 (7%)	5 (8%)	2 (7%)	
Adjuvant CT^b^				
Yes	86 (90%)	60 (90%)	26 (87%)	0.528
No	10 (10%)	6 (10%)	4 (13%)	
DFI (median, month) (range)	11 (0–100)	10 (0–70)	12 (1–100)	0.333
Triger for detecting mTNBC				
Some symptoms	35 (36%)	17 (26%)	18 (60%)	0.005
Routine examinations	36 (38%)	29 (44%)	7 (23%)	
Not documented	25 (26%)	20 (30%)	5 (17%)	
Performance status				
0/1	83 (86%)	66 (100%)	17 (57%)	< 0.001
2	5 (5%)	0 (0%)	5 (17%)	
3/4	8 (8%)	0 (0%)	8 (27%)	
Number of metastatic sites^a^ (mean) (range)	1.4 (1–4)	1.4 (1–4)	1.4 (1–3)	0.970
Visceral metastasis				
Yes	59 (61%)	41 (62%)	18 (60%)	0.843
No	37 (39%)	25 (38%)	12 (40%)	
Metastatic site				
Bone	18 (19%)	15 (23%)	3 (10%)	0.139
Liver	24 (25%)	14 (21%)	10 (33%)	0.204
Lung	40 (42%)	28 (42%)	12 (40%)	0.823
Brain	15 (16%)	4 (6%)	11 (37%)	< 0.001
Others	39 (41%)	32 (48%)	7 (23%)	0.020

^a^At the time of diagnosis for distant metastasis. ^b^Including neoadjuvant chemotherapy. IDC: invasive ductal carcinoma; NST: no special type; CT: chemotherapy; DFI: disease-free interval.

In 25 patients, the trigger for the discovery of mTNBC was not documented because these cases dated back to the paper-based medical record era, in which detailed documentation was not routinely preserved. Therefore, we next compared the same clinicopathological factors for the remaining 71 cases for which information was complete, according to the presence or absence of symptoms at the time of mTNBC diagnosis (Supplementary Material 2, wjon.elmerpub.com). There were some trends in metastatic organs. Brain metastases were significantly more common in the symptomatic group, while liver metastases were more common in the asymptomatic group (P = 0.003 and P = 0.011, respectively). Among the patients with brain metastases in the symptomatic group, symptom status was available for 11 cases; 10 were diagnosed after the onset of neurological symptoms, and only one subsequently received systemic therapy, whereas the remaining nine did not receive any anticancer treatment. The higher prevalence of “other organs” in the symptomatic group is understandable, given that this group included several soft tissue metastases, such as subcutaneous masses. Meanwhile, no differences were observed between the two groups regarding factors such as pathological stage of the primary tumor and tumor grade.

### Clinicopathological features related to OS for mTNBC patients

Next, clinicopathological factors associated with survival after diagnosis of mTNBC in the treated group (n = 66) were examined. The median OS was 14 months (range: 1–94 months); 59 died of breast cancer and seven were still on treatment. Univariate analysis revealed that age, number of metastatic organs at mTNBC diagnosis, presence of brain metastasis, and administration of PTX + BVZ were significantly associated with OS (P = 0.023, P = 0.007, P = 0.016, and P = 0.028, respectively) (Table 2). In multivariate analysis, “number of metastatic organs” (hazard ratio (HR) = 8.88; 95% confidence interval (CI), 1.2–50.2; P = 0.020) and “administration of PTX + BVZ” (HR = 0.44; 95% CI, 0.2–0.9; P = 0.029) were associated with OS. Accordingly, a shorter OS was observed in patients with a higher number of metastatic organs at mTNBC diagnosis, whereas PTX + BVZ administration was associated with longer OS in the multivariable analysis. Meanwhile, factors such as younger age and brain metastases, which were observed to be associated with shorter OS in univariate analysis, were not significantly associated after multivariable adjustment (P = 0.240 and P = 0.739, respectively). Additionally, baseline NLR was not associated with OS.

**Table 2:** Relationship Between Clinicopathological Features and OS (N = 66)

Variables	Univariate			Multivariate		
	HR	95% CI	P value	HR	95% CI	P value
Age^a^	0.33^b^	0.1–0.9	0.023	0.41^b^	0.1–1.8	0.240
Histology, NST vs. others	0.88	0.4–2.1	0.768			
High tumor grade, yes vs. no	1.68	0.9–2.9	0.062	1.03	0.5–2.0	0.925
HER2, low vs. null	0.92	0.5–1.6	0.762			
Adjuvant CT, yes vs. no	1.98	0.8–4.6	0.119			
DFI (months)	0.46^b^	0.1–1.5	0.210	0.90^b^	0.2–4.3	0.894
Number of metastatic sites^a^	4.81^b^	1.6–13.0	0.007	8.88^b^	1.2–50.2	0.020
Visceral metastasis^a^, yes vs. no	1.25	0.7–2.2	0.422			
Metastatic site						
Bone	1.55	0.8–2.9	0.158			
Liver	1.29	0.7–2.5	0.438			
Lungs	0.93	0.6–1.6	0.794			
Brain	3.66	1.3–10.5	0.016	0.74	0.1–4.3	0.739
ALC^a^	0.46^b^	0.1–2.0	0.321			
NLR^a^	1.35^b^	0.5–3.4	0.544	1.14^b^	0.4–3.3	0.811
Administration of ICI, yes vs. no	0.70	0.3–1.8	0.444			
Administration of PTX + BVZ, yes vs. no	0.54	0.3–0.9	0.028	0.44	0.2–0.9	0.029

^a^At the time of diagnosis for distant metastasis. ^b^The range odds ratio. OS: overall survival; HR: hazard ratio; CI: confidence interval; NST: no special type; CT: chemotherapy; DFI: disease-free interval; ALC: absolute lymphocyte count; NLR: neutrophil-to-lymphocyte ratio; ICI: immune checkpoint inhibitor-based treatment; PTX: paclitaxel; BVZ: bevacizumab.

**Figure 2:**
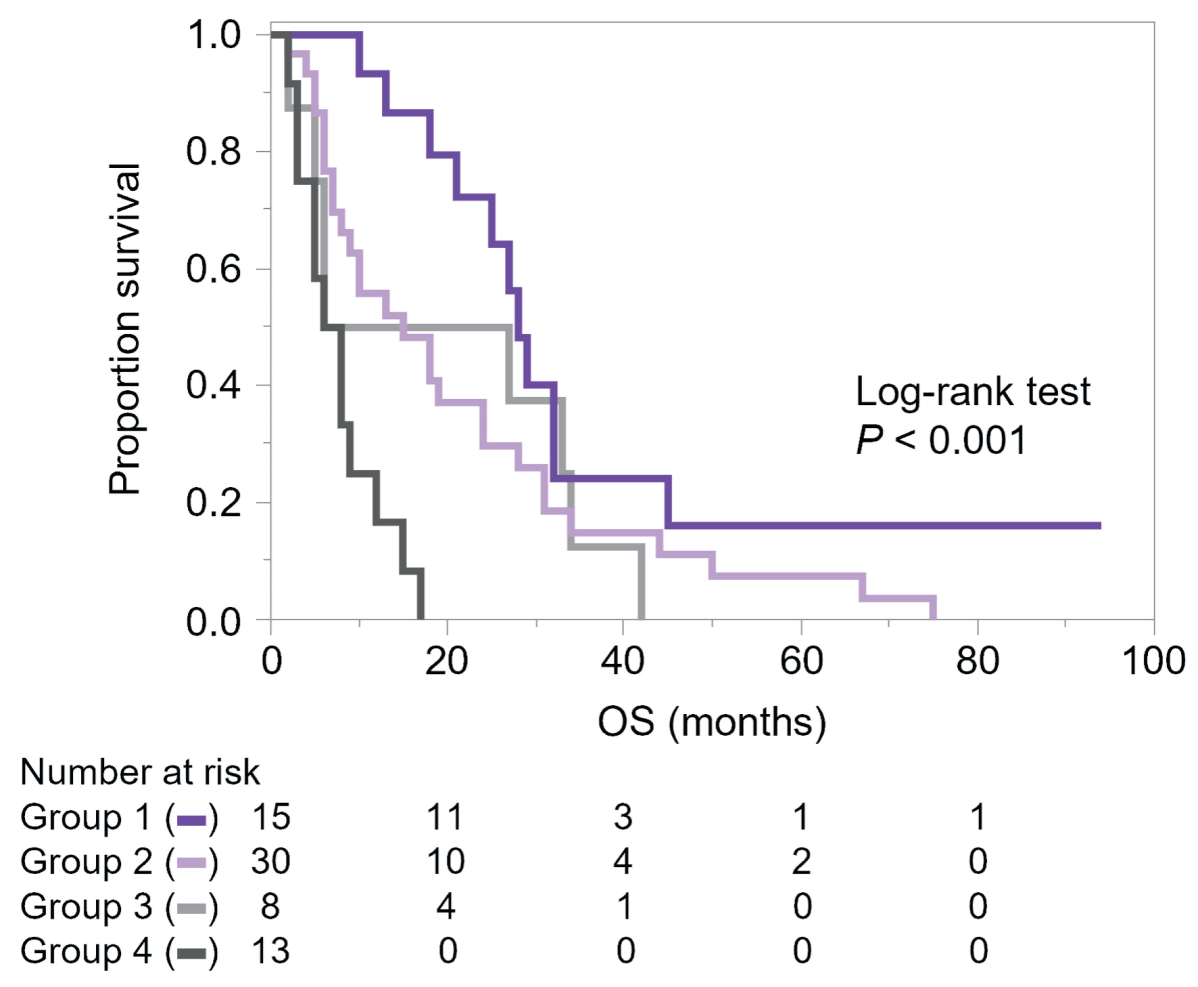
Kaplan–Meier curves of overall survival (OS) according to the number of metastatic organs and administration of paclitaxel (PTX) and bevacizumab (BVZ). Metastatic organs were counted numerically without weighting for organ-specific prognostic impact. Group 1 (purple): solitary metastasis with PTX + BVZ (n = 15); group 2 (light purple): solitary metastasis without PTX + BVZ (n = 30); group 3 (light grey): multiple metastases with PTX + BVZ (n = 8); and group 4 (grey): multiple metastases without PTX + BVZ (n = 13).

Kaplan–Meier curves were then drawn according to a combination of the number of metastatic organs and PTX + BVZ administration, which were associated with OS in exploratory analyses. In this analysis, metastatic sites were counted numerically without weighting for organ-specific prognostic impact; and the number of metastatic organs was divided into two groups: patients with only one organ with metastases and those with two or more, considering the mean value of 1.4 for the number of metastatic organs (Fig. 2). As a result, we confirmed that patients who developed multiple metastases at baseline and did not receive PTX + BVZ had the shortest OS. On the other hand, patients who developed solitary metastasis at the time of mTNBC diagnosis and were treated with PTX + BVZ at some point had the longest OS.

### Details of systemic drug treatment for mTNBC patients

**Figure 3:**
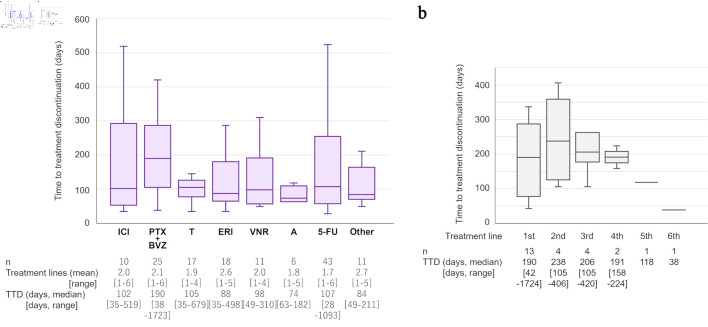
Analysis of treatment lines and time to treatment discontinuation. (a) Details and comparisons of systemic drug treatments for metastatic triple-negative breast cancer are shown. “Other” includes gemcitabine (n = 7), mitomycin C plus methotrexate (n = 1), CMF (cyclophosphamide, methotrexate, and fluorouracil; n = 1), olaparib (n = 1), and trastuzumab–deruxtecan (n = 1). (b) Time to treatment discontinuation (TTD) of PTX + BVZ according to treatment line are shown. ICI: immune checkpoint inhibitor-based treatment; PTX: paclitaxel; BVZ: bevacizumab; T: taxanes; ERI: eribulin; VNR: vinorelbine; A: anthracycline; 5-FU: 5-fluorouracil.

As this was a retrospective observational study, the treatment of individual patients in the treated group varied among patients. Therefore, the duration of each treatment was analyzed in detail. The mean number of treatment lines for the 66 patients was 2.2 (range: 1–6). Among various treatments, PTX + BVZ showed the longest median TTD (190 days; range: 38–1,723 days) (Fig. 3a), and was longer than those of anthracycline-based and other treatments. Meanwhile, oral 5-FU was the most commonly used drug, as 43 of 66 patients (65%) received this treatment. ICI-based treatment, which included atezolizumab plus nab-PTX and pembrolizumab plus carboplatin and gemcitabine regimens, were only given to 10 of 66 patients (15%), as this was relatively recently introduced to the clinic. As an additional analysis, the TTD of PTX + BVZ was further examined according to the number of treatment lines for PTX + BVZ (Fig. 3b). There was no difference in median TTD between the first to the fourth line.

Finally, we focused on 5-FU, which in some patients had a very long TTD, and investigated the background factors associated with TTD (Supplementary Material 3, wjon.elmerpub.com). We divided the patients into two groups: long and short 5-FU, based on the median TTD of 5-FU treatment (105.5 days). We found that the 5-FU treatment was longer in older patients and patients with a lower tumor grade (P = 0.014 and P = 0.049, respectively). On the other hand, no association was observed with the number of metastatic organs or the presence of visceral metastases at the time of 5-FU administration.

## Discussion

According to our real-world study, in 31% of mTNBC patients, their general condition at diagnosis was poor, or the disease had progressed too far, preventing proper drug therapy. Many patients in the non-treated group were diagnosed with mTNBC after presenting with symptoms. Predicting mTNBC before the onset of symptoms was difficult, as there were no significant differences in clinicopathological features such as pathological stage of the primary tumor or tumor grade between the two groups with and without symptoms. Since brain metastases were more frequently observed in the symptomatic group, consistent with a previous report [16], it remains crucial to identify predictive factors for tumors that metastasize to the brain in TNBC or to establish methods for early detection of brain metastases.

Routine imaging or blood tests for detecting distant metastases are not recommended in the guidelines for follow-up after curative surgery for breast cancer. Several randomized trials conducted in the 1990s, which form the basis of today’s guidelines, showed that routine postoperative surveillance, including imaging (such as chest X-ray, bone scintigraphy, chest and abdominal computed tomography, etc.) and blood tests, did not improve survival rates [17, 18], although led to earlier detection of recurrence. However, with the introduction of more effective targeted therapies for recurrent breast cancer, such as BVZ and ICIs, there may be room for improving postoperative follow-up strategies now. In our cohort, asymptomatic metastases were usually detected during postoperative follow-up imaging or laboratory examinations, which were performed at the discretion of treating physicians rather than by standardized surveillance protocols. This context should be considered when interpreting the outcomes associated with asymptomatic diagnosis. In the current study, two-thirds of the treated group were diagnosed with mTNBC and started treatment while still asymptomatic. On the other hand, more than half of the patients in the non-treated group were diagnosed after symptoms appeared, had a higher prevalence of poor performance status, and were unable to receive appropriate treatment and eventually died. Considering this, there is a need to reconsider postoperative surveillance, taking into account factors such as stage and subtype. In Japan, a prospective trial is currently underway by the Japan Clinical Oncology Group to evaluate the significance of intensive postoperative surveillance in high-risk patients (JCOG1204; UMIN000012429) [19]. Patients in the intensive surveillance group will be monitored postoperatively for 5 years using chest and abdominal computed tomography, bone scintigraphy, and head magnetic resonance imaging/computed tomography. Since hormone receptor status and HER2 status are included as stratification factors, the results will be interpretable by subtype, and the outcomes are eagerly awaited.

It was unsurprising that the greater the number of metastatic organs at the time of mTNBC diagnosis, the shorter the OS. Using the Surveillance, Epidemiology, and End Results (SEER) database, Wang et al analyzed the prognosis of over 2,000 stage IV TNBC patients according to metastatic organ [20]. In their analysis, the prognosis for patients with multiple metastases was particularly poor, and having multiple metastases contributed more to a poor prognosis than having brain metastases. Our data fully aligned with the findings observed in that study. On the other hand, in our current study, NLR at the time of mTNBC diagnosis was not correlated with OS. While NLR is an established prognostic marker for poor outcomes in both primary and recurrent breast cancer [21, 22], this study exclusively focused on patients with mTNBC. It is possible that NLR was relatively high in all patients, which may explain why it is less effective as a prognostic marker for OS in mTNBC.

In our cohort, an association with longer OS was observed in patients who received the PTX + BVZ regimen. The combination therapy of BVZ with chemotherapy has been shown to significantly prolong progression-free survival in several phase III trials for HER2-negative metastatic breast cancer [23, 24]. While these trials did not show an improvement in OS, some studies, such as the subgroup analysis of the RIBBON-2 trial focusing on TNBC, have suggested a benefit in OS [25, 26]. The combination of PTX and BVZ was widely indicated as first-line treatment for adult patients with metastatic breast cancer, including in Europe, the UK and Korea, in addition to Japan, until the introduction of ICIs. However, in this retrospective cohort, because PTX + BVZ was administered at different time points during the disease course, immortal time bias cannot be excluded, and the observed association with longer OS should be interpreted with caution. While many patients in our cohort were treated before the introduction of ICIs, it is almost certain that the utility of ICIs will be established for PD-L1-positive patients, whose prognosis is poorer without immunotherapy [27, 28], in RWD in the near future. However, for PD-L1-negative patients, there is still no clear first-line treatment option available. On the other hand, our findings on PTX + BVZ may provide useful information for selecting second-line treatments and beyond. Even though ICI-based treatment has now been established as a primary option, the subsequent course of treatment still remains unclear [1, 2, 29]. It is also notable that a number of mTNBC patients continued oral 5-FU for an extended period, with treatment durations exceeding 3 years in some cases. Such prolonged treatment may reflect the underlying tumor biology, including factors like low tumor grade. Our data confirm the importance of detailed biological analysis of TNBC to advance personalized treatment [1, 2, 10].

One limitation of this study is the variability in postoperative follow-up and treatment strategies among patients, which is inherent to retrospective observational studies. The sample size was also limited, reducing the statistical power and preventing comparisons before and after the introduction of ICI-based treatment. In addition, given the limited sample size relative to the number of covariates, the full-model multivariable Cox analysis should be interpreted as exploratory, and model instability or overfitting cannot be excluded. Therefore, validation in larger cohorts and comparisons with second-generation agents, such as sacituzumab govitecan, will be necessary. In addition, analyses of the associations between PD-L1 status and recurrence patterns, metastatic organ involvement, and treatment outcomes were not feasible because PD-L1 expression was unknown in most cases. In addition, the treated and non-treated groups represented clinically distinct situations, making statistical matching methods unsuitable. Nevertheless, our findings revealed a considerable group of patients who experienced rapid progression and became ineligible for treatment at the time of recurrence detection. Identifying such patients earlier remains a major challenge and requires prospective evaluation and case accumulation.

### Conclusions

Employing RWD, we observed that numerous mTNBC cases had already progressed significantly at the time of diagnosis, limiting the opportunity to receive appropriate drug therapy. This finding suggests the importance of optimizing follow-up systems to enable earlier therapeutic intervention. To achieve early diagnosis, it is necessary to integrate existing blood tests and imaging modalities, and to develop and evaluate novel biomarkers, particularly those capable of predicting or detecting brain metastases at an early stage. In addition, treatment strategies beyond second-line therapy warrant further investigation, including clarification of the potential role of paclitaxel plus bevacizumab (PAC + AVA) and descriptive evaluation of the feasibility of maintenance therapy using oral agents in biologically low-grade cases. To expand treatment opportunities and improve long-term outcomes, it is essential to prospectively evaluate follow-up strategies and biomarkers that enable the diagnosis of mTNBC before symptomatic deterioration.

## Supplementary Material

Suppl 1 Comparisons in adjuvant chemotherapy regimens between the treated and the non-treated groups.

Suppl 2 Clinicopathological factors according to presence or absence of symptoms at the time of diagnosis.

Suppl 3 Relationship between oral 5-FU treatment duration and clinicopathological features (n = 40).

## Data Availability

All data supporting the findings of this study are available within the paper and its supplementary information.

## Abbreviations

5-FU: 5-fluorouracil
BVZ: bevacizumab
CI: confidence interval
CT: computed tomography
ER: estrogen receptor
ERI: eribulin
HER2: human epidermal growth factor receptor 2
ICI: immune checkpoint inhibitor
IQR: interquartile range
JCOG: Japan Clinical Oncology Group
KM: Kaplan–Meier
MRI: magnetic resonance imaging
mTNBC: metastatic triple-negative breast cancer
NLR: neutrophil-to-lymphocyte ratio
OS: overall survival
PAC + AVA: paclitaxel plus bevacizumab
PD-L1: programmed death-ligand 1
PTX: paclitaxel
RWD: real-world data
SEER: Surveillance, Epidemiology, and End Results
SG: sacituzumab govitecan
T-DXd: trastuzumab deruxtecan
TNBC: triple-negative breast cancer
TTD: time to treatment discontinuation
VNR: vinorelbine
