# Supplementary material for: Clinical Outcomes and Prognostic Factors in Metastatic Triple-Negative Breast Cancer: A Real-World Data Analysis
Source: World J Oncol. 2026 Mar 5;17(2):268–76. doi: 10.14740/wjon2713 (PMC12978387; doi:10.14740/wjon2713)
Supplement: Suppl 1 — Comparisons in adjuvant chemotherapy regimens between the treated and the non-treated groups. [file wjon-17-02-268-s001.docx]

**Suppl 1.** Comparisons in adjuvant chemotherapy regimens between the treated and the non-treated groups

| Adjuvant chemotherapy regimen | Total | Treated group (n = 60) | Non-treated group (n = 26) | *P* value |
| --- | --- | --- | --- | --- |
| Anthracycline + Taxane | 68 | 52 (87%) | 16 (62%) |  |
| Anthracycline alone | 9 | 4 (7%) | 5 (19%) | 0.003 |
| Taxane alone | 5 | 4 (7%) | 1 (4%) |  |
| Oral fluoropyrimidine (5-FU) | 4 | 0 (0%) | 4 (15%) |  |
