# Supplementary material for: Clinical Outcomes and Prognostic Factors in Metastatic Triple-Negative Breast Cancer: A Real-World Data Analysis
Source: World J Oncol. 2026 Mar 5;17(2):268–76. doi: 10.14740/wjon2713 (PMC12978387; doi:10.14740/wjon2713)
Supplement: Suppl 2 — Clinicopathological factors according to presence or absence of symptoms at the time of diagnosis. [file wjon-17-02-268-s002.docx]

**Suppl 2.** Clinicopathological factors according to presence or absence of symptoms at the time of diagnosis

IDC; invasive ductal carcinoma, NST; no special type, CT; chemotherapy, DFI; disease-free interval

^a^At the time of diagnosis for distant metastasis, ^b^Including neoadjuvant chemotherapy
