# Supplementary material for: Clinical Outcomes and Prognostic Factors in Metastatic Triple-Negative Breast Cancer: A Real-World Data Analysis
Source: World J Oncol. 2026 Mar 5;17(2):268–76. doi: 10.14740/wjon2713 (PMC12978387; doi:10.14740/wjon2713)
Supplement: Suppl 3 — Relationship between oral 5-FU treatment duration and clinicopathological features (n = 40). [file wjon-17-02-268-s003.docx]

**Suppl 3.** Relationship between oral 5-FU treatment duration and clinicopathological features (n=40)

| Variables |  | Long 5-FU | Short 5-FU | *P* value |
| --- | --- | --- | --- | --- |
| n |  | 20 | 20 |  |
| Age^a^ (mean) |  | 63.2 | 52.1 | 0.014 |
| Histology | NST | 17 (85%) | 19 (95%) | 0.282 |
|  | Others | 3 (15%) | 1 (5%) |  |
| High tumor grade | Yes | 6 (32%) | 12 (63%) | 0.049 |
|  | No | 13 (68%) | 7 (37%) |  |
| HER2 | Low | 13 (68%) | 12 (60%) | 0.583 |
|  | Nul | 6 (32%) | 8 (40%) |  |
| Number of metastatic sites^a^ (mean) |  | 1.1 | 1.4 | 0.103 |
| Visceral metastasis^a^ | Yes | 10 (50%) | 15 (75%) | 0.103 |
|  | No | 10 (50%) | 5 (25%) |  |
| Previous administration of PTX+BVZ | Yes | 5 (25%) | 2 (10%) | 0.212 |
|  | No | 15 (75%) | 18 (90%) |  |

NST; no special type, PTX; paclitaxel, BVZ; bevacizumab

^a^At the time of administration of 5-FU treatment.
